# Supplementary material for: Comparative outcomes of DSAEK and DMEK in eyes with prior Ahmed glaucoma valve implantation
Source: PLoS One. 2026 Mar 2;21(3):e0344183. doi: 10.1371/journal.pone.0344183 (PMC12952605; doi:10.1371/journal.pone.0344183)
Supplement: Attachment — Submitted filename: PLOS one_ DMEK Vs DSAEK.docx [file pone.0344183.s001.docx]

**Manuscript ID:** PONE-D-25-33955
**Title:** Comparative Outcomes of DSAEK and DMEK in Eyes with Prior Ahmed Glaucoma Valve Implantation

This is a well written retrospective study comparing DSAEK and DMEK in eyes with prior AGV implantation. This is a clinically valuable and addressing below points will further strengthen its impact.

1. Please clarify how the surgical approach (DSAEK vs. DMEK) was selected. Since DMEK was performed by a single surgeon and DSAEK by two others, could surgeon experience have influenced outcomes?
2. Donor age and ECD were significantly different between groups. Was donor tissue allocated randomly or preferentially? Please explain how donor assignment was handled and whether any donor-related selection bias is possible.
3. Given that ultrathin DSAEK was used (mean graft thickness ~58 µm), please discuss how this may have influenced the comparability to DMEK outcomes. Prior literature shows thinner DSAEK grafts may yield outcomes closer to DMEK.
4. The reported rebubbling rates were relatively high. Were any specific protocols used for air tamponade or postoperative positioning to account for the presence of AGV? If so, please include these details.
5. Was AGV tube position (e.g., angle, anterior chamber, sulcus) recorded and analyzed? This factor may influence graft detachment and endothelial trauma and could explain some postoperative variability.
6. Were systemic comorbidities such as DM or autoimmune conditions equally distributed between groups? These factors may affect corneal healing and graft survival and should be addressed if data are available.
7. Please confirm whether all eligible cases during the study period were included and how selection bias was minimized. Was data extraction blinded to the surgical group or outcome?
8. Given the comparable outcomes, do the authors recommend either technique in resource-limited settings where donor quality or surgical experience may vary?
9. The authors report similar graft survival despite prior AGV placement. How do the authors reconcile this with studies that report lower graft survival in AGV eyes? Please discuss possible protective or confounding factors in this cohort.
