# Supplementary material for: Comparative outcomes of DSAEK and DMEK in eyes with prior Ahmed glaucoma valve implantation
Source: PLoS One. 2026 Mar 2;21(3):e0344183. doi: 10.1371/journal.pone.0344183 (PMC12952605; doi:10.1371/journal.pone.0344183)
Supplement: Attachment — Submitted filename: PLOS ONE_Response_letter_1st revised_final.docx [file pone.0344183.s004.docx]

Manuscript ID: PONE-D-25-33955
Title: Comparative Outcomes of DSAEK and DMEK in Eyes with Prior Ahmed Glaucoma Valve Implantation

Reviewer #1:

Authors described a comparative study in terms of clinical outcomes after two types of endothelial keratoplasty following AGV. Despite the interesting topic, there are some concerns to be raised.

1) Authors should show the statistic power using Gpower to justify the number they used.

- We appreciate your insightful comments that have significantly strengthened our manuscript. We agree this is an important consideration. Due to limited eligible cases, the study recruited 16 and 11 eyes in groups DSAEK and DMEK, respectively. This sample size inherently limits statistical power. With these group sizes, a two-sided α = 0.05 test has 80% power to detect only very large standardized differences (Cohen’s d ≈ 1.15), as calculated by G*Power.

- We added “Mean Difference (95% CI)” values to all comparative tables to present the absolute magnitude and uncertainty of group differences more clearly. We also submitted Hedges’ g values (small sample correction of Cohen’s d) for each parameter to show standardized effect sizes adjusted for small sample bias as a supplement file.

- We have added power analysis to the Limitations section: "Due to limited eligible cases, a two-sided α=0.05 test has 80% power to detect only very large standardized differences (Cohen's d ≈ 1.15) (calculated by G*power). Therefore, the lack of significant differences should be interpreted cautiously."(lines 263-266). Additionally, we have also revised our Results and Discussion to use more cautious language when interpreting our findings.

2) Although they criticize previous studies due to heterogeneity of etiologies, the current study include various glaucoma. Especially, the number of PEX looks different between two groups. Due to small numbers, there is no significant difference.

We acknowledge this concern. While our cohort includes various glaucoma diagnoses, we have several important points, the distribution of glaucoma types was statistically comparable between groups (P=0.545), including PEX (DSAEK: 2 cases [12.5%] vs DMEK: 2 cases [18.2%]). All eyes shared the common feature of AGV-induced endothelial decompensation, which was our primary focus. This is now emphasized in the Discussion (lines 192-201). We believe that our study's strength lies in focusing exclusively on a single type of drainage device (AGV), despite the heterogeneity of etiologies.

3) Authors should add the presence of PAS(peripheral anterior synechiae) in detail in all cases. The existence of PAS might affect the survival rates.

- We completely agree that PAS is a clinically important factor. Unfortunately, due to the retrospective nature of this study, detailed PAS documentation was not consistently available in all medical records. We have now acknowledged this limitation explicitly (lines 270-273): "Third, certain clinically relevant factors such as peripheral anterior synechiae, iris defects, and other anterior chamber comorbidities could not be assessed. These factors may influence surgical outcomes, particularly in complex cases with prior glaucoma surgery." We have also added Schoenberg et al.'s reference (Ref. 27), which demonstrated that high DSAEK failure rates in AGV eyes are associated with 360° PAS.

4) Authors should add the lens status in detail in pseudophakic cases. How many scleral fixated IOLs, or sulcus fixated IOLs were included?

- We thank the reviewer for this helpful suggestion. We have now described the lens status in pseudophakic eyes in detail in Table 1, including the number of in-the-bag IOLand scleral-fixated IOLs (There were no eyes with the sulcus placed IOL).

5) There is no information about the axial length, or ACD(anterior chamber depth).

- We thank the reviewer for this helpful suggestion. Axial length and anterior chamber depth have now been added in Table 1.

6) Authors should describe the location of implanted Ahmed tube in all cases. How many tubes behind iris, anterior chamber, and pars plana?

- We thank the reviewer for this important comment. We have clarified this important surgical detail. All 27 eyes had AGV tubes placed in the anterior chamber. None were placed in the posterior chamber (behind iris) or pars plana. This is now explicitly stated in the Results section (line 123):"All implanted AGV tubes were placed in the anterior chamber."

7) Authors should describe the special technique during endothelial keratoplasty in terms of trimming the tips of tubes in the AC, or not.

- Excellent observation. Two patients underwent Ahmed valve trimming during endothelial keratoplasty, while valve repositioning was not necessary in the other cases. We have added detailed information about valve tip management in both the Results and Complications sections (lines 167-172): "In two DMEK cases, valve tip trimming was required during surgery, because the valve tip could potentially interfere with DMEK graft unfolding procedure. One of these patients subsequently underwent valve tip repositioning into the sulcus due to uncontrolled IOP elevation and iris chafing. Nevertheless, graft survival was favorable, with a ECD >1000 cells/mm² at postoperative month 12, comparable to the overall cohort."

8) Discussion
Authors seem to conclude that younger age is only a predictor of graft survival.
Please read the paper, and do consider the discussion again.

Kitazawa K, Toda M, Ueno M, Uehara A, Sotozono C, Kinoshita S. The Biologic
Character of Donor Corneal Endothelial Cells Influences Endothelial Cell Density
Post Successful Corneal Transplantation. Ophthalmol Sci. 2022 Oct
28;3(2):100239. doi: 10.1016/j.xops.2022.100239. PMID: 36846106; PMCID:
PMC9944567.

- Thank you for this important reference. We now acknowledge that donor age is likely a surrogate for underlying cellular biological properties rather than a direct causal factor. We have carefully reviewed Kitazawa et al.'s paper and substantially revised our Discussion to incorporate these insights (lines 227-232, Ref 17): "However, recent evidence suggests that donor age may serve as a surrogate marker, with intrinsic biological characteristics of donor endothelial cells, including cellular senescence and mitochondrial function, representing the true determinants of postoperative ECD and long-term graft outcomes.[17] Future studies controlling for multiple donor-related confounding factors are warranted to comprehensively elucidate which donor characteristics most critically influence graft survival and longevity in eyes with glaucoma drainage devices."

Reviewer #2:

This is a well written retrospective study comparing DSAEK and DMEK in eyes with prior AGV implantation. This is a clinically valuable and addressing below points will further strengthen its impact.

1. Please clarify how the surgical approach (DSAEK vs. DMEK) was selected. Since DMEK was performed by a single surgeon and DSAEK by two others, could surgeon experience have influenced outcomes?
- We thank the reviewer for this thoughtful comment. We acknowledge that surgeon experience might have influenced the outcomes. However, each surgeon selected their preferred surgical method, which we believe helped minimize bias arising from variability in surgical proficiency. This point has been addressed in the Methods (line 91-93) and Discussion section (line 267-270) as a potential limitation.

2. Donor age and ECD were significantly different between groups. Was donor tissue allocated randomly or preferentially? Please explain how donor assignment was handled and whether any donor-related selection bias is possible.
- We thank the reviewer for raising this important point. The donor age difference was not due to preferential allocation but reflects inherent technical requirements of each procedure. We have extensively explained this in the Discussion (lines 218-223): " This age distribution reflects differing technical requirements between the procedures. For DMEK, donors aged 55-70 years are generally preferred because younger donor tissue exhibits greater Descemet membrane elasticity, causing tighter graft scrolling that complicates both preparation and anterior chamber unfolding. In contrast, DSAEK can accommodate a wider donor age range, including younger tissue, as the stromal component provides structural support facilitating handling. "

3, Given that ultrathin DSAEK was used (mean graft thickness ~58 µm), please discuss how this may have influenced the comparability to DMEK outcomes. Prior literature shows thinner DSAEK grafts may yield outcomes closer to DMEK.

- We agree with the reviewer that the relatively thin grafts used in our UT-DSAEK group (mean thickness ~58 µm) could have influenced comparability with DMEK. Prior literature has consistently shown that ultrathin DSAEK approaches the visual outcomes of DMEK, with thinner grafts associated with better visual acuity and lower higher-order aberrations compared with conventional DSAEK. Therefore, our UT-DSAEK results may partially reflect this convergence toward DMEK outcomes, which should be taken into account when interpreting the findings. We have added substantial discussion of this important factor (lines 206-211): "Additionally, we performed ultrathin DSAEK (mean graft thickness: 58.14 ± 11.88 μm, range: 45–88 μm), which uses a much thinner graft than conventional DSAEK. Previous studies demonstrated that ultrathin DSAEK achieved better visual outcomes than conventional DSAEK, with results comparable to DMEK.[13,14] These factors may explain the comparable visual outcomes between DSAEK and DMEK observed in our study."

4. The reported rebubbling rates were relatively high. Were any specific protocols used for air tamponade or postoperative positioning to account for the presence of AGV? If so, please include these details.

- We did not employ any specific modifications in surgical protocols for air tamponade or postoperative management in eyes with AGV. However, in order to maintain the intracameral air bubble position and to prevent premature escape of air, patients were more strictly instructed to remain in the supine position immediately after surgery. We mentioned this in Methods section. The rebubbling rates (25% DSAEK, 27% DMEK) are within the reported range for eyes with GDDs (15-33%), as we discuss in lines 236-241.

5. Was AGV tube position (e.g., angle, anterior chamber, sulcus) recorded and analyzed? This factor may influence graft detachment and endothelial trauma and could explain some postoperative variability.

- We thank the reviewer for this important comment. In all cases, the AGV tube was in the anterior chamber. This is now explicitly stated in the Results section (line 123):"All implanted AGV tubes were placed in the anterior chamber."

6. Were systemic comorbidities such as DM or autoimmune conditions equally distributed between groups? These factors may affect corneal healing and graft survival and should be addressed if data are available.

- We thank the reviewer for this important comment. We have added systemic comorbidities that could be listed in the chart to Table 1: Diabetes mellitus: DSAEK 3 (18.8%), DMEK 2 (18.2%), Hypertension: DSAEK 6 (37.5%), DMEK 3 (27.3%), Autoimmune disease: DSAEK 1 (6.3%), DMEK 0 (0%); There were no statistically significant differences between the two groups (P=1.000).

7. Please confirm whether all eligible cases during the study period were included and how selection bias was minimized. Was data extraction blinded to the surgical group or outcome?

- We thank the reviewer for this important comment. All eligible cases with well-documented follow-up records for at least 12 months after surgery were included in the analysis. All consecutive AGV cases meeting inclusion criteria during the study period were included, with no exclusions based on surgical outcomes. To minimize selection bias, data extraction was performed in a blinded manner with respect to both surgical group and outcomes. We have added this important methodological detail to Methods (lines 88-90): "Data extraction and analysis were performed by independent observers who were not involved in surgical procedures or clinical care to minimize selection bias."

8. Given the comparable outcomes, do the authors recommend either technique in resource-limited settings where donor quality or surgical experience may vary?

- We thank the reviewer for this helpful suggestion. Considering that our study demonstrated comparable outcomes, we agree that surgical and resource factors should guide the choice in resource-limited settings. In resource-limited settings, DSAEK may offer advantages due to: wider donor age acceptability (our DSAEK donors ranged 27-66 years vs DMEK 55-88 years), potentially easier learning curve with more forgiving surgical technique, comparable outcomes when using ultrathin grafts. However, DMEK remains excellent when appropriate donor tissue and surgical expertise are available. Since it was not possible to generalize the experiences and conditions of individual practitioners, we only noted that both are viable options for AGV eyes. We address this in our revised Conclusion (lines 278-280):". These findings suggest that both procedures appear to be viable options for corneal endothelial decompression in eyes with AGV and that the choice between them can be based on surgeon preference, experience, and donor tissue availability. "

9. The authors report similar graft survival despite prior AGV placement. How do the authors reconcile this with studies that report lower graft survival in AGV eyes? Please discuss possible protective or confounding factors in this cohort.

- Our 2-year survival rates (78% for DSAEK, 74% for DMEK) are notably lower than those reported in uncomplicated cases (90-96%), confirming that AGV presence significantly compromises graft survival. Our rates fall within the range reported by other GDD studies (66-82% at 2 years), rather than exceeding them. Importantly, we do not claim that our cohort had unexpectedly good outcomes or protective factors against AGV-related graft failure. Rather, our findings show that: Both techniques demonstrated the expected lower survival in AGV eyes compared to uncomplicated cases. Within this high-risk population, the two techniques performed comparably to each other. Both techniques remain viable options when managing this challenging patient population.
