# Supplementary material for: Comparative outcomes of DSAEK and DMEK in eyes with prior Ahmed glaucoma valve implantation
Source: PLoS One. 2026 Mar 2;21(3):e0344183. doi: 10.1371/journal.pone.0344183 (PMC12952605; doi:10.1371/journal.pone.0344183)
Supplement: Attachment — Submitted filename: Peer review of PONE-D-25-33955.docx [file pone.0344183.s003.docx]

**Peer review of “Comparative Outcomes of DSAEK and DMEK in Eyes with Prior Ahmed Glaucoma Valve 2 Implantation”**

This study describes the visual and graft outcomes of DSAEK and DMEK in eyes that bear an Ahmed Glaucoma Valve. Despite its small sample size, it adds to the literature, which is limited to a few studies on DMEK alone or DSAEK alone in glaucoma drainage device (GDD) cases or studies that compare the two techniques but do not distinguish between trabeculotomy and GDD. The paper is generally well-written, although some key aspects were not clearly indicated. However, the most important aspects of keratoplasty (visual outcome, endothelial cell loss, perioperative complications, and graft survival) were described.

# Major points

1. The DMEK patients had thicker preoperative CCTs than the DSAEK patients (838 vs 775 µm). This suggests that the extent of edema was worse in the DMEK patients. Intense and/or prolonged edema can lead to stromal fibrosis, which can significantly limit visual recovery after DMEK or DSAEK. Thus, the equivalent outcomes of DMEK and DSAEK in the present study may reflect worse bullous keratopathy and stromal fibrosis in the DMEK cases. It is possible that the visual outcomes would have been better in DMEK if disease severity in the two groups was equivalent. This should be discussed.
2. In relation to this, it is suggested in the Discussion that UT-DSAEK can achieve equivalent visual outcomes as DMEK. This is actually a very contested area, with as many studies showing that DMEK outperforms UT-DSAEK (e.g. the RCTs in doi:10.1097/ICO.0000000000002601 and doi:10.1016/j.ophtha.2018.05.019, and the fellow DMEK:DSAEK eye studies in doi:10.1155/2015/750567 and doi:10.1016/j.ophtha.2020.12.021). Lines 206-209 should be phrased more cautiously.
3. The Methods should explictly indicate how glaucoma diagnosis was made and what categories were included (POAG, etc).
4. The Methods should explictly state that cases with 1 or ≥2 AGV and mixed AGV:trabeculectomy/MIGs were included.
5. The Methods should make clear that the patients were “all consecutive patients” and that no DMEK/DSAEK eyes with AGV were excluded for any reason.
6. The postoperative ECD data should also be expressed as %ECL. It is a commonly used measure that accounts for preoperative ECD.
7. The fact that the statistical comparisons in Table 4 did not detect the thicker CCT in DSAEK (due to the extra stroma in the graft) demonstrates the risk of false negatives with small sample sizes. In a larger cohort, this difference would be statistically significant. This point should be included in the Limitations section.
8. Line 167 “In two DMEK cases, valve tip trimming was required during surgery, because the valve tip could potentially interfere with DMEK graft unfolding procedure”.

How was it determined that the valve tip could interfere with DMEK unfolding? Was it after the DMEK graft was introduced into the AC? Or can such interference be detected before the procedure? If the latter, DSAEK may be preferable?

9. There was patient attrition due to graft failure (5 DSAEK and 3 DMEK eyes). When these graft failures occurred during the 12-month study period should be shown with a patient distribution figure. This attrition will also have affected the eye numbers in Tables 2-4. How many eyes were left at the various timepoints should be indicated in these tables.
10. How DSAEK and DMEK in the study compare to uncomplicated DSAEK/DMEK should be briefly described in the Discussion. Does the presence of AGV drastically hamper visual recovery, induce higher ECL, and reduce graft survival?
11. A brief description of how the study findings compare to previous studies (refs 8-12) is needed. A table summarizing the key features of these studies can help with this.

# Minor points:

1. Line 73 “This retrospective study analyzed patients who visited at Seoul St.Mary’s Hospital 28/07/2022 and 27/07/2023 and underwent EK at Seoul St. Mary's Hospital between 01/01/2020 and 31/12/2022.”

The relevance of the first-cited but later visit (between 28/07/2022 and 27/07/2023) is unclear. Only the period when the actual surgery was conducted is relevant. The follow-up timepoints (eg. Day 1, Month 1 etc) should also be explictly stated in the Methods.

2. Line 81 “Medical records were reviewed for slit-lamp findings, best-corrected visual acuity (BCVA), intraocular pressure (IOP), surgical history, ocular and systemic comorbidities, ECD, CCT, and postoperative complications, including rebubbling, IOP elevation, and cystoid macular edema (CME).”

When postoperative BCVA, ECD, CCT, IOP, graft attachment, and the macula were examined should be indicated here. The instruments used for these measurements and their manufacturers should also be indicated. How AXL and ACD were measured should also be indicated.

3. Line 86 “Secondary graft failure was defined as irreversible corneal edema following initial improvement.”

This should be defined more precisely. Eg “Secondary graft failure was defined as the reemergence of corneal edema, after initial improvement, that arose at any time point during the 12-month study period.”

4. Line 87 “Postoperative IOP elevation was assessed based on the need for additional glaucoma medications and an IOP exceeding 21 mm Hg.”

Does this relate to only the first week? The postoperative period relating to this should be specified.

5. Line 93 “Any specific modifications were employed.”

The meaning of this was unclear. Please clarify.

6. Line 93 “For 94 DSAEK, precut donor tissue with a graft thickness less than 100 µm was obtained from a nonprofit eye bank” & Line 104 “For DMEK, precut and preloaded grafts in bent-pipette glass tubes were obtained from a nonprofit eye 105 bank”

How were the grafts stored and transported? Which medium, which temperature?

7. Line 96 “Following viscoelastic injection, Descemet's membrane was scored along the circular mark and carefully stripped using a modified reverse Sinskey 98 hook. Following viscoelastic injection, Descemet's 97 membrane was scored along the circular mark and carefully stripped using a modified reverse Sinskey 98 hook.”

The manufacturer details of the viscoelastic and hook should be provided.

8. Line 107 “Filtered room air was used for graft attachment, followed by 2 to 4 hours of supine positioning. Patients were strictly instructed to remain in the supine position and avoid eye rubbing after surgery. Rebubbling with filtered room air or 20% sulfur hexafluoride was performed when at least one-third of the graft was found to be detached postoperatively”

Why was SF6 used for rebubbling but not the endotamponade? It lasts a lot longer than air, generally one would use it for endotamponade, not for rebubbling grafts that are already partially attached.

9. Were the 3 phakic cases left phakic, or was the triple procedure conducted? This should be specified in the Methods.
10. The degree of graft detachment that was considered to indicate the need for rebubbling should be defined. Generally 30% area detached and/or central detachment are considered indications for rebubbling.
11. Line 169 “One of these patients subsequently underwent valve tip repositioning into the sulcus due to 170 uncontrolled IOP elevation and iris chafing.”

When during the 12-month follow up did this occur?

12. Were the uncontrolled IOP episodes all controlled eventually?
13. Line 198 “all eyes shared the common feature of AGV-induced endothelial decompensation”.

This should be phrased more cautiously, eg “all eyes exhibited endothelial decompensation, likely due to the AGV”

14. Line 202 “...contrast with previous 203 reports in uncomplicated cases, where DMEK typically demonstrates superior visual recover”.

References are needed for this statement.

15. Line 219 “For DMEK, donors aged 55-70 years are generally preferred because younger donor tissue exhibits greater Descemet membrane elasticity, causing tighter graft scrolling that complicates both preparation and anterior chamber unfolding.”

References are needed for this statement.

16. Line 219 “For DMEK, donors aged 55-70 years are generally preferred because younger donor tissue 220 exhibits greater Descemet membrane elasticity, causing tighter graft scrolling that complicates both preparation and anterior chamber unfolding.”

It should be indicated here that these complications induce ECL, which in turn promotes graft failure.

17. Line 226 “Previous studies have demonstrated associations between younger donor age and superior endothelial cell survival following corneal transplantation15,16.”

Refs 15 and 16 relate to DSAEK and PKP. It is likely that selecting older donors for DMEK – which leads to less graft handling and ECL - counterbalances the effect of older donor age. This should be indicated here.

18. Line 238 “The graft detachment/rebubbling rate is difficult to conclude”

This is unclear and should be clarified.

19. Line 267 “However, our study reduced the risk of selection bias,”

Briefly summarize here how your study design reduced the risk of selection bias.

20. Line 274 “Despite these limitations, our study provides meaningful contributions to clinical practice.”

This should be more cautious eg “our study may be useful for keratoplasty surgeons dealing with eyes bearing an AGV.”

# Typos

1. Line 67: “This study aimed to address this knowledge gap by comparing the 68 outcomes of DSAEK and DMEK in eyes with single type of GDD devices, or AGVs.”

This should read “...with a single type of GDD, namely, AGVs”

1. Line 112 “Mann-Whiteny U test”
1. The AGV ≥2 data are not on the same row as the row title in Table 1.
2. Line 132 “All values are presented as mean ± standard deviation. percentage (%).”
3. Line 194 “we specifically focused on eyes with a single type of glaucoma drainage device, or AGV,”

This should read “eyes with a single type of GDD, namely, AGV,”

4. Line 265 “Therefore, the lack of significant differences should be interpreted cautiously. s potential confounders.
6. Line 270 “population Third,”
7. Line 278 “These findings suggest that both procedures appear to be viable options for corneal endothelial decompression in eyes with AGV”

Decompression should be decompensation.

8. Quotation mark at Line 285.
