# Supplementary material for: Comparative outcomes of DSAEK and DMEK in eyes with prior Ahmed glaucoma valve implantation
Source: PLoS One. 2026 Mar 2;21(3):e0344183. doi: 10.1371/journal.pone.0344183 (PMC12952605; doi:10.1371/journal.pone.0344183)
Supplement: Attachment — Submitted filename: Response to Reviewer_2nd revision.docx [file pone.0344183.s005.docx]

**Response to Reviewer**

We sincerely thank the reviewer for the careful re-evaluation of our revised manuscript and for the insightful and constructive comments. We have carefully addressed all points raised and believe that the manuscript has been substantially strengthened as a result. Below, we provide a point-by-point response.

**Major Points**

**1.The DMEK patients had thicker preoperative CCTs than the DSAEK patients (838 vs 775 µm). This suggests that the extent of edema was worse in the DMEK patients. Intense and/or prolonged edema can lead to stromal fibrosis, which can significantly limit visual recovery after DMEK or DSAEK. Thus, the equivalent outcomes of DMEK and DSAEK in the present study may reflect worse bullous keratopathy and stromal fibrosis in the DMEK cases. It is possible that the visual outcomes would have been better in DMEK if disease severity in the two groups was equivalent. This should be discussed.**

**Response:**
We agree with the reviewer that the greater preoperative CCT in the DMEK group may reflect more advanced corneal edema and potentially greater stromal fibrosis, which could limit postoperative visual recovery. We have now explicitly addressed this important point in the Discussion and acknowledged baseline disease severity as a potential confounder that may have attenuated the visual advantage of DMEK.

**Manuscript change (Discussion):** “Secondly, preoperative central corneal thickness was numerically greater in the DMEK group than in the DSAEK group, suggesting more advanced corneal edema at baseline. Prolonged or severe corneal edema may induce stromal fibrosis, which can constrain postoperative visual recovery regardless of the endothelial keratoplasty technique.”

**2. In relation to this, it is suggested in the Discussion that UT-DSAEK can achieve equivalent visual outcomes as DMEK. This is actually a very contested area, with as many studies showing that DMEK outperforms UT-DSAEK (e.g. the RCTs in doi:10.1097/ICO.0000000000002601 and doi:10.1016/j.ophtha.2018.05.019, and the fellow DMEK:DSAEK eye studies in doi:10.1155/2015/750567 and doi:10.1016/j.ophtha.2020.12.021). Lines 206-209 should be phrased more cautiously.**

**Response:**
We appreciate this important clarification. We have revised the relevant sentences to adopt a more cautious tone and to clearly distinguish our findings in AGV-bearing eyes from evidence derived from uncomplicated cases and randomized controlled trials. We now emphasize that our findings should not be interpreted as equivalence in general, but rather as comparable outcomes in a highly specific and complex clinical context.

**Manuscript change (Discussion):**

“Taken together, these considerations may help explain the comparable visual outcomes observed in our study and should not be interpreted as evidence of equivalence between DSAEK and DMEK in uncomplicated eyes.”

**3. The Methods should explictly indicate how glaucoma diagnosis was made and what categories were included (POAG, etc).**

**Response:**
We agree and have clarified the diagnostic criteria and glaucoma subtypes included in the Methods section.

Manuscript change (Methods): “Glaucoma diagnosis was based on prior clinical diagnosis by glaucoma specialists and included primary open-angle glaucoma, secondary glaucoma, uveitic glaucoma, pseudoexfoliative glaucoma, neovascular glaucoma, and iridocorneal endothelial syndrome.”

**4. The Methods should explictly state that cases with 1 or ≥2 AGV and mixed AGV:trabeculectomy/MIGs were included.**

**Response:**
We have revised the Methods to explicitly state that eyes with single or multiple AGVs, as well as those with additional prior glaucoma surgeries, were included.

Manuscript change (Methods): “Eyes with a single AGV, multiple AGVs (≥2), and those with prior trabeculectomy or microinvasive glaucoma surgery in addition to AGV implantation were included.”

**5. The Methods should make clear that the patients were “All eligible cases were consecutively included, with no exclusions among eyes with prior AGV implantation undergoing DSAEK or DMEK.”**

**Response:**

We agree and have explicitly stated this in the Methods.

Manuscript change (Methods): “All eligible cases during the study period were consecutively included, and no eyes with prior AGV implantation undergoing DSAEK or DMEK were excluded for any reason.”

**6. The postoperative ECD data should also be expressed as %ECL. It is a commonly used measure that accounts for preoperative ECD.**

**Response:**
We agree with the reviewer and have additionally calculated postoperative endothelial cell loss as percentage endothelial cell loss (%ECL), using donor endothelial cell density as the reference. This measure was included to account for baseline donor ECD and to allow a complementary assessment of endothelial outcomes. A new table (Table 4) has been added to present %ECL values at each postoperative time point. As shown, %ECL did not differ significantly between the DSAEK and DMEK groups at any time point. Corresponding descriptions have been added to both the Methods and Results sections.

**Manuscript changes:**

Methods: “Postoperative endothelial cell loss (ECL) was additionally calculated and expressed as a percentage (%ECL) relative to the donor ECD using the following formula: %ECL = [(donor ECD − postoperative ECD) / donor ECD] × 100.”

Results: “Postoperative %ECL showed no statistically significant differences between the DSAEK and DMEK groups at 1, 3, 6, or 12 months postoperatively (Table 4).” A new table (Table 4) summarizing %ECL comparisons between DSAEK and DMEK was added.

**7.** **The fact that the statistical comparisons in Table 4 did not detect the thicker CCT in DSAEK (due to the extra stroma in the graft) demonstrates the risk of false negatives with small sample sizes. In a larger cohort, this difference would be statistically significant. This point should be included in the Limitations section.**

**Response:**
We agree with the reviewer and have explicitly addressed this point in the Limitations section.

**Manuscript change (Limitations):** “In this context, the lack of a significant difference in postoperative CCT, even with the inherent stromal thickness of DSAEK grafts, highlights the potential for false-negative findings in a small cohort.”

**8.** **Line 167 “In two DMEK cases, valve tip trimming was required during surgery, because the valve tip could potentially interfere with DMEK graft unfolding procedure”. How was it determined that the valve tip could interfere with DMEK unfolding? Was it after the DMEK graft was introduced into the AC? Or can such interference be detected before the procedure? If the latter, DSAEK may be preferable?**

**Response:**
We appreciate this request for clarification. Valve tube length was assessed intraoperatively before graft insertion. When the AGV tube was excessively long on intraoperative assessment, valve tip trimming was performed before graft insertion to avoid interference with graft positioning. This decision was based on the surgeon’s anatomical assessment rather than the choice of endothelial keratoplasty technique.

**Manuscript change (Methods):** “When the AGV tube was excessively long on intraoperative assessment, valve tip trimming was performed before graft insertion to avoid interference with graft positioning.”

**9.** **There was patient attrition due to graft failure (5 DSAEK and 3 DMEK eyes). When these graft failures occurred during the 12-month study period should be shown with a patient distribution figure. This attrition will also have affected the eye numbers in Tables 2-4. How many eyes were left at the various timepoints should be indicated in these tables.**

**Response:**
We thank the reviewer for this important comment and have clarified these points in the revised manuscript. The Kaplan–Meier survival curve (Figure 1) includes all graft failure events observed over the entire follow-up period, and the timing of all failures (5 DSAEK and 3 DMEK eyes) is now explicitly indicated in the figure, together with the number of remaining eyes at each time point.

The outcome analyses presented in Tables 2 – 5 (newly numbered tables) were designed from the outset to represent a 12-month cohort. At each postoperative visit, eyes that had experienced graft failure by that time point were excluded from the analysis. Accordingly, the number of eyes analyzed differed across time points and is now explicitly reported in the Results and in table footnotes (DSAEK/DMEK: 16/11 at 1 week, 1 month, and 3 months; 16/10 at 6 months; and 14/10 at 12 months).

**10.** **How DSAEK and DMEK in the study compare to uncomplicated DSAEK/DMEK should be briefly described in the Discussion. Does the presence of AGV drastically hamper visual recovery, induce higher ECL, and reduce graft survival?**

**Response:**
This comparison is briefly addressed in the Discussion. We noted that, unlike uncomplicated endothelial keratoplasty where DMEK typically demonstrates superior visual outcomes and higher graft survival, eyes with prior AGV implantation showed attenuated visual recovery and reduced graft survival regardless of the EK technique. These findings emphasize that the presence of an AGV represents a challenging anterior chamber environment compared with uncomplicated cases.

**11.** **A brief description of how the study findings compare to previous studies (refs 8-12) is needed. A table summarizing the key features of these studies can help with this.**

**Response:**
We appreciate the reviewer’s suggestion. Prior studies (refs 8–12) are already cited and discussed in the Discussion to contextualize our findings. We chose not to add a summary table because the referenced studies are highly heterogeneous, and presenting only selected studies in tabular form could introduce selection bias and imply a level of systematic review beyond the scope of this original research article.

**Minor points**

**1. Line 73 “This retrospective study analyzed patients who visited at Seoul St.Mary’s Hospital 28/07/2022 and 27/07/2023 and underwent EK at Seoul St. Mary's Hospital between 01/01/2020 and 31/12/2022.” The relevance of the first-cited but later visit (between 28/07/2022 and 27/07/2023) is unclear. Only the period when the actual surgery was conducted is relevant. The follow-up timepoints (eg. Day 1, Month 1 etc) should also be explictly stated in the Methods.**

**Response:**
We agree with the reviewer. The visit period was unclear and potentially confusing. We have removed this information and revised the Methods to specify only the surgical period. We have also explicitly stated the postoperative follow-up time points.

**Manuscript change (Methods):**

“This retrospective study included patients who underwent endothelial keratoplasty between 01/01/2020 and 31/12/2022, with postoperative evaluations performed at regular follow-up visits during the 12-month postoperative period.”

**2. Line 81 “Medical records were reviewed for slit-lamp findings, best-corrected visual acuity (BCVA), intraocular pressure (IOP), surgical history, ocular and systemic comorbidities, ECD, CCT, and postoperative complications, including rebubbling, IOP elevation, and cystoid macular edema (CME).” When postoperative BCVA, ECD, CCT, IOP, graft attachment, and the macula were examined should be indicated here. The instruments used for these measurements and their manufacturers should also be indicated. How AXL and ACD were measured should also be indicated.**

**Response:**
We appreciate this suggestion and have expanded the Methods to specify the timing, measurement devices, and manufacturers.

**Manuscript change (Methods):**

“Postoperative BCVA, IOP, ECD, and CCT were assessed at every visit throughout the 12-month postoperative period. BCVA was measured using a Snellen chart and converted to logMAR units. IOP was measured using a non-contact pneumotonometer (CT-80A; Canon Inc., Tokyo, Japan). ECD and CCT were assessed using specular microscopy (CellChek XL; Konan Medical, Nishinomiya, Japan) and an ultrasound pachymeter (SP-3000; Tomey Corporation, Nagoya, Japan), respectively. Axial length (AL) and anterior chamber depth (ACD) were measured preoperatively using optical biometry (IOLMaster 700; Carl Zeiss Meditec, Jena, Germany). Graft attachment was evaluated postoperatively using slit-lamp biomicroscopy and anterior segment optical coherence tomography (ANTERION; Heidelberg Engineering, Heidelberg, Germany). Retinal and macular status were evaluated at postoperative 1 and 3 using ultra-widefield imaging (Optos Silverstone; Optos PLC, Dunfermline, UK) and spectral-domain OCT (Heidelberg Retina Angiograph OCT; Heidelberg Engineering, Heidelberg, Germany).”

**3. Line 86 “Secondary graft failure was defined as irreversible corneal edema following initial improvement.”**

**This should be defined more precisely. Eg “Secondary graft failure was defined as the reemergence of corneal edema, after initial improvement, that arose at any time point during the 12-month study period.”**

**Response:**
We agree and have revised the definition to be more precise.

**Manuscript change (Methods):**

“Secondary graft failure was defined as the reemergence of corneal edema after initial postoperative improvement, occurring at any time during the 12-month follow-up period.”

**4.** **Line 87 “Postoperative IOP elevation was assessed based on the need for additional glaucoma medications and an IOP exceeding 21 mm Hg.”**

**Does this relate to only the first week? The postoperative period relating to this should be specified.**

**Response:**
Thank you for this clarification request. We have specified that postoperative IOP elevation was assessed throughout the entire follow-up period.

**Manuscript change (Methods):**

“Postoperative IOP elevation was defined as an IOP exceeding 21 mm Hg and/or the need for additional glaucoma medications at any time during the 12-month postoperative follow-up period.”

**5. Line 93 “Any specific modifications were employed.” The meaning of this was unclear. Please clarify.**

**Response:**
We agree that this statement was unclear. As no systematic technique modifications were applied beyond standard approaches, this phrase has been removed.

**6.** **Line 93 “For DSAEK, precut donor tissue with a graft thickness less than 100 µm was obtained from a nonprofit eye bank” & Line 104 “For DMEK, precut and preloaded grafts in bent-pipette glass tubes were obtained from a nonprofit eye bank” How were the grafts stored and transported? Which medium, which temperature?**

**Response:**
We have added details regarding graft storage and transport conditions.

**Manuscript change (Methods):**

“All donor corneal tissues were preserved in Optisol-GS and transported at 4°C according to standard eye bank protocols.”

**7.** **Line 96 “Following viscoelastic injection, Descemet's membrane was scored along the circular mark and carefully stripped using a modified reverse Sinskey hook. Following viscoelastic injection, Descemet's membrane was scored along the circular mark and carefully stripped using a modified reverse Sinskey hook.” The manufacturer details of the viscoelastic and hook should be provided.**

**Response:**
We have added the manufacturer information for the viscoelastic material (Hyalu®; Hanmi Pharm. Co., Ltd., Seoul, Korea) and a modified reverse Sinskey hook (Moria SA, Antony, France).

**8.** **Line 107 “Filtered room air was used for graft attachment, followed by 2 to 4 hours of supine positioning. Patients were strictly instructed to remain in the supine position and avoid eye rubbing after surgery. Rebubbling with filtered room air or 20% sulfur hexafluoride was performed when at least one-third of the graft was found to be detached postoperatively”**

Why was SF6 used for rebubbling but not the endotamponade? It lasts a lot longer than air, generally one would use it for endotamponade, not for rebubbling grafts that are already partially attached.

**Response:**
Both filtered room air and 20% sulfur hexafluoride are commonly used for initial graft tamponade as well as for rebubbling, depending on surgeon preference and intraoperative or postoperative considerations. In our practice, room air was routinely used for primary graft attachment, whereas either room air or 20% sulfur hexafluoride was selected for rebubbling at the surgeon’s discretion.

**9.** **Were the 3 phakic cases left phakic, or was the triple procedure conducted? This should be specified in the Methods.**

**Response:**
Thank you for raising this point. All phakic eyes were left phakic at the time of endothelial keratoplasty, and no simultaneous or subsequent cataract surgery was performed during the study period. This has now been clarified in the Methods section.

**Manuscript change (Methods):**

“All phakic eyes were left phakic at the time of DMEK or DSAEK, and no simultaneous or subsequent cataract surgery was performed during the study period.”

**10. The degree of graft detachment that was considered to indicate the need for rebubbling should be defined. Generally 30% area detached and/or central detachment are considered indications for rebubbling.**

**Response:**
We thank the reviewer for this comment. The criterion for rebubbling was already specified in the Methods section. As stated in the manuscript, rebubbling was performed when at least one-third of the graft was found to be detached postoperatively. We have now slightly revised the wording to further improve clarity.

**Manuscript change (Methods):**

“Rebubbling with filtered room air or 20% sulfur hexafluoride was performed when graft detachment involved at least one-third of the graft area postoperatively.”

**11. Line 169 “One of these patients subsequently underwent valve tip repositioning into the sulcus due to uncontrolled IOP elevation and iris chafing.”**

**Response:**
We have clarified that valve tip repositioning was performed at 4 months postoperatively.

**12.** **Were the uncontrolled IOP episodes all controlled eventually?**

**Response:**
Yes. We have clarified this outcome in the Results.

**Manuscript change (Results):**

“All other episodes of postoperative IOP elevation were successfully controlled with additional glaucoma medications.”

**13.** **Line 198 “all eyes shared the common feature of AGV-induced endothelial decompensation”. This should be phrased more cautiously, eg “all eyes exhibited endothelial decompensation, likely due to the AGV”**

**Response:**
We agree and have revised the wording to avoid causal overstatement.

**Manuscript change (Discussion):**

“All eyes exhibited endothelial decompensation, likely due to the AGV.”

**14.** **Line 202 “...contrast with previous reports in uncomplicated cases, where DMEK typically demonstrates superior visual recover”. References are needed for this statement.**

**Response:**
Appropriate references that were already included in the reference list have been added to support this statement (refs 13 and 14).

**15 & 16. Line 219 “For DMEK, donors aged 55-70 years are generally preferred because younger donor tissue exhibits greater Descemet membrane elasticity, causing tighter graft scrolling that complicates both preparation and anterior chamber unfolding.”**

**References are needed for this statement.**

**Line 219 “For DMEK, donors aged 55-70 years are generally preferred because younger donor tissue exhibits greater Descemet membrane elasticity, causing tighter graft scrolling that complicates both preparation and anterior chamber unfolding.”**

**It should be indicated here that these complications induce ECL, which in turn promotes graft failure.**

**Response:**
We thank the reviewer for these comments. We agree that this statement reflects a surgical principle based primarily on accumulated clinical experience rather than high-level comparative evidence. To support this widely accepted concept in the DMEK field, we have added an expert review that discusses the technical challenges associated with tightly scrolled DMEK grafts, which are more commonly encountered with younger donor tissue and may increase graft manipulation (new ref 15). This reference has been added to the reference list, and the reference numbering has been updated accordingly.

In addition, we have revised the text to clarify that increased graft manipulation during preparation and unfolding may contribute to endothelial cell loss, which can subsequently affect graft survival.

**17.** **Line 226 “Previous studies have demonstrated associations between younger donor age and superior endothelial cell survival following corneal transplantation15,16.”**

**Refs 15 and 16 relate to DSAEK and PKP. It is likely that selecting older donors for DMEK – which leads to less graft handling and ECL - counterbalances the effect of older donor age. This should be indicated here.**

**Response:**
We agree with the reviewer and have revised the Discussion to clarify that associations between younger donor age and endothelial cell survival reported in DSAEK and PKP may not directly apply to DMEK. We now indicate that, in DMEK, the use of older donor tissue may reduce graft manipulation and endothelial cell loss, potentially counterbalancing the effect of donor age, and that donor age may act as a surrogate marker for intrinsic endothelial cell characteristics.

**Manuscript change (Discussion):**

“Previous studies have demonstrated associations between younger donor age and superior endothelial cell survival following corneal transplantation16,17. However, this association may not directly apply to DMEK, in which the use of older donor tissue may reduce graft manipulation and ECL during procedures.”

**18.** **Line 238 “The graft detachment/rebubbling rate is difficult to conclude”**

**This is unclear and should be clarified.**

**Response:**
We have rephrased this sentence for clarity.

**Manuscript change (Discussion):**

“The interpretation of graft detachment and rebubbling rates is challenging, as these outcomes are influenced by multiple factors including surgical technique, anterior chamber anatomy, and the presence of glaucoma drainage devices.”

**19. Line 267 “However, our study reduced the risk of selection bias,”**

**Briefly summarize here how your study design reduced the risk of selection bias.**

**Response:**
We thank the reviewer for this comment. As described in the Methods section and in the Limitation of the Discussion section, the risk of selection bias was reduced by consecutively including all eligible eyes with prior AGV implantation that underwent DSAEK or DMEK during the study period, without excluding any cases based on clinical characteristics or outcomes. In addition, uniform inclusion criteria were applied, and each surgeon consistently performed a single endothelial keratoplasty technique, minimizing technique-related selection within each group.

**20.** **Line 274 “Despite these limitations, our study provides meaningful contributions to clinical practice.”**

**This should be more cautious eg “our study may be useful for keratoplasty surgeons dealing with eyes bearing an AGV.”**

**Response:**
We agree and have revised the sentence accordingly.

**Manuscript change:**

“Despite these limitations, our study may be useful for keratoplasty surgeons managing eyes with prior AGV implantation.”

**Typos**

All typographical and formatting errors listed by the reviewer have been corrected as suggested.
